# Predictive value and application of thrombelastography combined with coagulation parameters for adverse pregnancy outcomes

**DOI:** 10.12669/pjms.42.4.13510

**Published:** 2026-04

**Authors:** Yufang Zhang, Haiyan Wang, Yanwei Guo, Mengyuan Wang, Yanju Zhu

**Affiliations:** 1 Yufang Zhang, Department of Obstetrics and Gynecology, Affiliated Hospital of Chengde Medical College, Chengde 067000, Hebei, China; 2 Haiyan Wang, Department of Obstetrics and Gynecology, Affiliated Hospital of Chengde Medical College, Chengde 067000, Hebei, China; 3 Yanwei Guo, Department of Obstetrics and Gynecology, Affiliated Hospital of Chengde Medical College, Chengde 067000, Hebei, China; 4 Mengyuan Wang, Department of Obstetrics and Gynecology, Affiliated Hospital of Chengde Medical College, Chengde 067000, Hebei, China; 5 Yanju Zhu, Department of Obstetrics and Gynecology, Affiliated Hospital of Chengde Medical College, Chengde 067000, Hebei, China

**Keywords:** Adverse pregnancy outcome, Coagulation parameter, Predictive value, Thrombelastography

## Abstract

**Objective:**

To evaluate the predictive value and clinical utility of thrombelastography (TEG) combined with coagulation parameters for adverse pregnancy outcomes(APOs).

**Methodology:**

A retrospective analysis was conducted on the clinical data of 186 pregnant women admitted to the Department of Obstetrics in Affiliated Hospital of Chengde Medical College between January 2022 and June 2025. Based on pregnancy outcomes, patients were classified into the APO group and the normal pregnancy outcome group. Baseline characteristics, TEG parameters, and coagulation parameters were compared between groups. Pearson correlation analysis was performed to assess associations between TEG and coagulation parameters in the APO group.

**Results:**

No significant differences were observed between the two groups in baseline characteristics such as age, gravidity, parity, and gestational weight gain (all P > 0.05), indicating comparability. Regarding TEG parameters, the APO group showed lower R values and higher maximum amplitude (MA) and coagulation index (CI) values compared with the normal outcome group (P < 0.05, respectively). For coagulation parameters, fibrinogen (FIB) and D-dimer (D-D) levels were significantly elevated, while antithrombin III (AT-III) levels were decreased in the APO group (P < 0.05, respectively).

**Conclusion:**

The combination of TEG and coagulation parameters demonstrates higher predictive efficiency for APOs than either method alone, providing a reliable basis for the early identification of high-risk populations and guiding clinical intervention strategies.

## INTRODUCTION

Fetal growth restriction, placental abruption, and postpartum hemorrhage are among the major adverse pregnancy outcomes (APOs), representing a critical public health challenge in maternal and child health worldwide. According to the latest statistics from the World Health Organization, APOs account for more than 15% of perinatal deaths annually on a global scale, while complications such as preeclampsia and placental abruption contribute to approximately 23%-35% of obstetric intensive care admissions for severe maternal conditions.^1^,^2^ Identifying precise, effective methods for early prediction to achieve risk stratification and timely intervention in APOs remains an urgent issue in obstetric medicine.

During pregnancy, the maternal system undergoes a physiological hypercoagulable state in order to adapt to the potential risk of hemorrhage at delivery. Although this hemostatic adjustment provides a protective mechanism against postpartum bleeding, it may simultaneously disrupt the balance between coagulation and anticoagulation, thereby predisposing to pathological thrombus formation. Previous studies^3^ have demonstrated that local placental microthrombosis constitutes a common pathological basis for APOs such as fetal growth restriction and placental abruption. This suggests that coagulation parameters may serve as important predictors of APOs. However, conventional coagulation tests, such as prothrombin time (PT) and D-dimer (D-D), reflect only isolated steps within the coagulation cascade and therefore present certain limitations. PT, for example, primarily reflects plasma coagulation factor activity but fails to assess platelet function, the kinetics of fibrin formation, or fibrinolytic activity.

Although D-D can indicate thrombus formation and fibrinolytic activation, its diagnostic specificity is limited, as physiologic hypercoagulability in late pregnancy often leads to false positives. This makes it difficult to distinguish between physiological and pathological coagulation abnormalities, restricting these parameters in guiding precise early intervention.^4^,^5^ Thrombelastography (TEG), as a novel technique for global hemostasis assessment, simulates the in vivo coagulation process and continuously monitors the entire course from initial clot formation to fibrinolysis. This allows comprehensive evaluation of coagulation factor activity, platelet aggregation, fibrin formation capacity, and fibrinolytic function, thereby enabling quantitative assessment of overall coagulation status.^6^,^7^ To date, evidence regarding the predictive value of TEG combined with conventional coagulation parameters for APOs remains limited, and it is unclear whether their combined application can further improve predictive performance. Against this backdrop, this study retrospectively analyzed the clinical data of 186 pregnant women admitted to the Department of Obstetrics in Affiliated Hospital of Chengde Medical College between January 2022 and June 2025 and evaluated the predictive value and clinical applicability of TEG in combination with coagulation parameters for APOs. The findings are expected to provide a scientific basis for individualized risk assessment and the development of early intervention strategies in clinical practice.

## METHODOLOGY

A retrospective analysis was conducted on the clinical data of 186 pregnant women who were hospitalized in the Department of Obstetrics in Affiliated Hospital of Chengde Medical College between January 2022 and June 2025. Participants data including demographic data were retrieved from electronic medical record systems from our hospital, and they were classified into two groups according to pregnancy outcomes: the APOs group (*n =* 40) and the normal pregnancy outcome(NPO) group(*n=* 146). APOs included fetal growth restriction, intrauterine fetal distress, placental abruption, preterm birth, postpartum hemorrhage, and postpartum deep vein thrombosis.^8^

### Ethical approval:

The study was approved by the Institutional Ethics Committee of Affiliated Hospital of Chengde Medical College (No.:CYFYLL2023527; Date: November 26, 2023), and written informed consent was obtained from all participants.

### Inclusion criteria:

- Singleton pregnancy.
- Completion of TEG and coagulation testing before initiation of anticoagulant therapy.
- Obtained the informed consent of the family.
- Availability of complete clinical data, including maternal age, gestational age, and pregnancy outcomes.

### Exclusion criteria:

- Multiple pregnancies or a history of adverse obstetric outcomes.
- Concurrent hepatic or renal insufficiency, autoimmune disease, thrombosis, or other disorders known to significantly affect coagulation.
- Pregnancy termination due to fetal chromosomal abnormalities or major congenital malformations.
- Surgery, acute infection, major trauma, or use of medications with significant effects on coagulation within one month prior to testing.

Clinical data were extracted and organized from the hospital electronic medical record system by the same investigator. Baseline data included maternal age, gravidity, parity, gestational weight gain, gestational age at delivery, body mass index (BMI) at delivery, and mode of delivery. TEG parameters included reaction time (R), maximum amplitude (MA), coagulation time(K), coagulation index (CI), and α-angle. Coagulation parameters included thrombin time (TT), activated partial thromboplastin time (APTT), PT, fibrinogen (FIB), D-D, and antithrombin III (AT-III).

Following clinical data collection, data entry was subjected to biweekly random sampling and verification (20% of cases per round). Consistency between entered data and original medical records was checked, and any inconsistent cases were excluded from analysis.

### Statistical analysis:

All data were analyzed using SPSS 26.0. Continuous variables were expressed as mean ± standard deviation (*x̅*+*s*), and their distribution was assessed using the Kolmogorov-Smirnov test. For normally distributed variables, comparisons between groups were performed using independent-samples *t* tests or one-way analysis of variance. For non-normally distributed variables, nonparametric tests were applied. Categorical variables were expressed as frequency and percentage (*n*[%]), and comparisons across groups were conducted using rank-sum tests. Pearson correlation analysis was used to evaluate associations, with an absolute correlation coefficient (|*r*|) > 0.3 considered indicative of a correlation. The Cox proportional hazards regression model was applied to identify risk factors. Predictive performance was assessed using receiver operating characteristic (ROC) curves, and the DeLong test was used to compare the area under the curve (AUC). A *P*-value <0.05 was considered statistically significant.

## RESULTS

No significant differences were observed in baseline variables, such as maternal age, gravidity, parity, gestational weight gain, BMI at delivery, or gestational age at delivery between the APO and NPO groups (all *P >* 0.05), indicating comparability between the two groups Table-I.

**Table-I:** Comparison of baseline characteristics between the APO and NPO groups.

Baseline data	APO group (n = 40)	NPO group (n = 146)	t/Z-value	P-value
Age (years)	29.00±3.20	28.50±3.10	0.898	0.371
Gravidity (times)	2.53±0.20	2.50±0.18	0.912	0.363
Parity (times)	1.10±0.20	1.05±0.25	1.166	0.245
Gestational weight gain (kg)	12.00±3.20	11.85±3.20	0.263	0.793
BMI at delivery (kg/m²)	28.20±1.95	28.07±1.89	0.383	0.702
Gestational age at delivery (weeks)	36.00±2.00	36.50±2.25	1.274	0.204
Mode of delivery, *n*(%)			3.690	0.158
Vaginal delivery	22(55.00)	85(58.22)		
Cesarean section	16(40.00)	60(41.10)		
Emergency cesarean	2(5.00)	1(0.68)		

Regarding TEG parameters, the APO group exhibited significantly lower R values and higher MA and CI values compared with the NPO group (*P <* 0.05, respectively). For coagulation parameters, the APO group showed significantly higher levels of FIB and D-D, and lower levels of AT-III, compared with the NPO group (*P <* 0.05, respectively) Table-II.

**Table-II:** Comparison of TEG and coagulation parameters between the APO and NPO groups.

Clinical data	APO group (n = 40)	NPO group (n = 146)	t-value	P-value
***TEG parameters***				
R (min)	5.20±0.52	7.50±0.85	16.280	<0.001
MA (mm)	68.20±4.20	60.50±5.28	8.509	<0.001
K (min)	1.18±0.35	1.24±0.39	0.880	0.380
CI	3.10±0.50	2.00±0.23	20.032	<0.001
α (°)	65.00±6.20	64.50±6.08	0.459	0.647
***Coagulation parameters***				
TT (s)	15.00±4.20	15.50±4.35	0.649	0.517
APTT (s)	29.20±5.10	29.00±5.02	0.222	0.824
PT (s)	10.80±1.50	11.00±1.95	0.601	0.548
FIB (g/L)	5.20±0.80	3.02±0.67	17.461	<0.001
D-D (mg/L)	1.50±0.30	0.82±0.14	20.507	<0.001
AT-III (%)	87.00±5.00	105.00±6.20	16.906	<0.001

Pearson correlation analysis revealed that in the APO group, the R value was negatively correlated with D-D (*r =* -0.402, *P <* 0.05). MA was positively correlated with both FIB (*r =* 0.558, *P <* 0.05) and D-D (*r =* 0.505, *P <* 0.05). CI was positively correlated with FIB (*r =* 0.492, *P <* 0.05) and D-D (*r =* 0.526, *P <* 0.05), while negatively correlated with AT-III (*r =* -0.357, *P <* 0.05) Table-III.

**Table-III:** Correlation between TEG and coagulation parameters in the APO group.

Parameter	FIB	D-D	AT-III
R	r = 0.156, P > 0.05	r = -0.402, P < 0.05	r = -0.237, P > 0.05
MA	r = 0.558, P < 0.05	r = 0.505, P < 0.05	r = -0.252, P > 0.05
CI	r = 0.492, P < 0.05	r = 0.526, P < 0.05	r = -0.357, P < 0.05

Using the occurrence of APOs (yes = 1, no = 0) as the dependent variable, parameters showing significant between-group differences in Table-II were included as independent variables in a Cox proportional hazards regression model with forward stepwise selection. The analysis identified R (hazard ratio [HR] [95% confidence interval, 95% CI] = 1.802 [1.065-2.539]), MA (HR [95% CI] = 1.752 [1.076-2.429]), CI (HR [95% CI] = 1.914 [1.065-2.762]), FIB (HR [95% CI] = 1.826 [1.000-2.652]), D-D (HR [95% CI] = 1.919 [1.025-2.814]), and AT-III (HR [95% CI] = 1.808 [1.068-2.547]) as independent risk factors for APOs (all *P <* 0.05) Table-IV.

**Table-IV:** Independent risk factors for APOs.

Variable	β	SE	Wald ^2^	P-value	HR (95%CI)
R	0.589	0.275	4.587	<0.001	1.802 (1.065-2.539)
MA	0.561	0.265	4.482	<0.001	1.752 (1.076-2.429)
CI	0.649	0.267	5.908	<0.001	1.914 (1.065-2.762)
FIB	0.602	0.265	5.161	<0.001	1.826 (1.000-2.652)
D-D	0.652	0.263	6.146	<0.001	1.919 (1.025-2.814)
AT-III	0.592	0.272	4.737	<0.001	1.808 (1.068-2.547)

**Fig.1:**
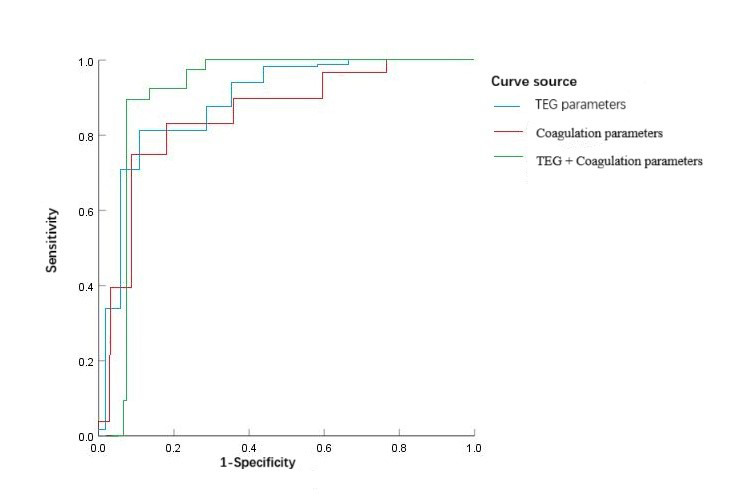
ROC curves for predicting APOs using TEG parameters, coagulation parameters, and their combination.

ROC curve analysis showed that the predictive efficiency of TEG parameters combined with coagulation parameters for APOs was superior to either method alone. The AUC for the combined model was 0.935, significantly higher than that for TEG parameters alone (AUC = 0.854) and coagulation parameters alone (AUC = 0.832) (*Z =* 2.972 and 3.016, both *P <* 0.05). Fig.1 and Table-V.

**Table-V:** ROC curve parameters for predicting APOs.

Test model	AUC	95% CI		Sensitivity (%)	Specificity (%)
		Lower	Upper		
TEG parameters	0.854	0.716	0.914	84.20	72.20
Coagulation parameters	0.832	0.705	0.902	82.60	70.50
TEG + Coagulation parameters	0.935	0.761	0.978	92.50	78.20

## DISCUSSION

In this study, Cox proportional hazards regression analysis demonstrated that both TEG parameters (R, MA, and CI) and coagulation parameters (FIB, D-D, and AT-III) were independent risk factors for APOs. These findings suggest that women with APOs present with a significant hypercoagulable state and an imbalance in the coagulation-fibrinolysis system, providing clinically applicable thresholds for early risk stratification. From the perspective of risk contribution, the R value (HR *=* 1.802) indicates that for every unit decrease in R, the risk of APOs increases by 80.2%. This may be attributed to the fact that a shortened R value directly reflects accelerated activation of coagulation factors. Excessive activation of these factors can lead not only to placental microthrombus formation but also to the activation of inflammatory pathways, thereby exacerbating endothelial injury and creating a vicious cycle of coagulation-inflammation, which in turn elevates the risk of APOs.^9^,^10^ Similarly, an elevated MA value (HR*=* 1.752) was associated with increased risk of APOs, primarily through mechanisms related to excessive clot strength. MA reflects the maximum strength or firmness of a clot formed through platelet-fibrin interactions; an elevated MA value indicates enhanced platelet-fibrin cross-linking.^11^ Such a hypercoagulable state may aggravate placental microcirculatory disturbances and increase resistance in uterine spiral arteries, directly reducing placental perfusion and ultimately leading to fetal distress, preterm birth, and other APOs. Among the TEG parameters, CI showed the highest HR (1.914), suggesting that it may provide the strongest predictive value for APOs. Using CI and other parameters, such as R and MA, can provide a more comprehensive reflection of coagulation imbalance.^12^

A CI value greater than 1.0 indicates a dual hypercoagulable state characterized by both accelerated initiation of coagulation and increased clot strength. Under these conditions, the risks of placental thrombosis and endothelial injury are significantly elevated, suggesting that CI may serve as a core indicator for APOs risk stratification. Among the conventional coagulation parameters, FIB (HR *=* 1.826), D-D (HR *=* 1.919), and AT-III (H*R =* 1.808) further refine the risk profile for APOs. Elevated FIB indicates an abundance of coagulation substrate, providing the material basis for thrombus formation. Increased D-D reflects not only activation of coagulation but also inadequate fibrinolytic clearance of formed thrombi, leading to their persistence and aggravation of tissue ischemia. Reduced AT-III implies compromised anticoagulant activity, resulting in insufficient suppression of excessively activated coagulation factors. These three parameters constitute a pathophysiological “risk chain” for APOs, spanning the dimensions of substrate sufficiency, coagulation activation, and weakened anticoagulation.^13^,^14^

This study further demonstrated significant correlations between TEG parameters and coagulation parameters in pregnant women with APOs. These correlations essentially represent a dynamic mapping of the pathological process involving coagulation activation, weakened anticoagulation, and compensatory fibrinolysis, thereby providing valuable reference indicators for early clinical identification and intervention. From a biological perspective, the R value, an index reflecting the interval from coagulation factor activation to initial fibrin formation, showed a negative correlation with D-D (*r =* -0.402, *P <* 0.05).

This finding corroborates the pathological logic that insufficient anticoagulation exacerbates accelerated coagulation initiation. In APOs, placental ischemia and inflammatory responses commonly trigger the coagulation cascade, enhance coagulation factor activity, and consequently shorten the R value.^15^ D-D, as a marker of fibrinolytic activation, may rise in parallel with coagulation activation, suggesting a coupled relationship in which stronger coagulation activation elicits more robust fibrinolytic compensation, thereby explaining the observed negative correlation. MA was positively correlated with FIB and D-D (*r =* 0.558 and 0.505, both *P <* 0.05), demonstrating the hypercoagulable intensity characteristic of APOs.

While elevated FIB in late pregnancy is considered a physiological phenomenon,^16^ in pregnant women with APOs, stress responses such as placental thrombosis associated with fetal growth restriction and endothelial injury in preeclampsia may lead to further elevation of FIB levels. The resulting high-resistance thrombi exacerbate placental microcirculatory disturbances. D-D, as a fibrinolytic by-product, showed a positive correlation with MA, indicating that increased clot strength is closely linked to activation of the fibrinolytic system. This finding suggests that disruption of the dynamic equilibrium between coagulation and fibrinolysis may simultaneously increase the risks of thrombosis and bleeding.^17^ The CI value serves as a quantitative indicator of the overall balance between coagulation and fibrinolysis, with elevated CI denoting a systemic hypercoagulable state.^18^

In this study, CI was positively correlated with FIB and D-D (*r =* 0.492 and 0.526, both *P <* 0.05) and negatively correlated with AT-III(*r=* -0.357, *P<* 0.05). These results indicate that increased coagulation substrate, activated coagulation-fibrinolysis pathways, and reduced anticoagulant activity jointly drive hypercoagulability. Essentially, CI consolidates disparate coagulation abnormalities into a single quantifiable hypercoagulable signal, offering a simplified yet effective reference for rapid identification of high-risk APOs in clinical practice. It is noteworthy that the correlations between TEG parameters and coagulation parameters were of only moderate strength (all |*r*| < 0.6). This may reflect the multifactorial nature of APOs, in which not only coagulation dysfunction but also endothelial impairment, inflammatory responses, and other factors are involved. Future work should consider integrating TEG, coagulation parameters, and endothelial biomarkers to construct multidimensional predictive models for more accurate risk stratification of APOs.

Coagulation parameters quantify the activity of specific blood components but capture only isolated aspects of the coagulation mechanism, failing to reproduce the dynamic and continuous nature of the hemostatic process. In contrast, TEG parameters simulate the in vivo coagulation cascade, capturing complex interactions among multiple components and their final effects, but they lack specificity for pinpointing abnormalities in individual factors. In this exploratory study, we integrated TEG parameters with coagulation parameters and found that their combined predictive value for APOs was superior (AUC= 0.935) compared with TEG parameters alone (AUC= 0.854) or coagulation parameters alone (AUC= 0.832) (*Z=* 2.972 and 3.016, both *P <* 0.05). The core value of this finding lies in overcoming the limitations of a single modality by establishing complementarity between dynamic and static assessments: TEG parameters provide continuous, process-level insights into abnormalities in coagulation initiation, clot strength, and global hemostatic balance, while conventional parameters offer static component-level information that clarifies the underlying causes of these abnormalities. This “process + component” model has the potential to guide more precise clinical interventions.^19^,^20^ For example, concurrent findings of shortened R, elevated CI, and reduced AT-III would suggest that the risk of APOs stems primarily from insufficient anticoagulant activity, indicating that AT-III supplementation may be a targeted intervention strategy.

### Limitations:

This study also has some shortcomings, such as a small number of samples were included and a single research indicator was conducted. On the basis of this study, we will continue to do large-scale sample and long-term follow-up studies to improve the credibility of the conclusion and the level of evidence.

## CONCLUSIONS

Combining TEG parameters with coagulation parameters yields greater predictive accuracy for APOs than either approach alone, providing a reliable basis for the early identification of high-risk populations and for tailoring individualized intervention strategies in clinical practice.

### Authors’ Contributions:

**YZ** and **HW:** Study design, literature search and manuscript writing, and are responsible and accountable for the accuracy or integrity of the work.

**YG** and **MW:** Data collection, data analysis and interpretation.

**YZ:** Were involved in the manuscript revision and validation.

All authors have read and approved the final manuscript.
